# Are our actions matching our words? A review of trainee ethnic and gender diversity in orthopaedic surgery

**DOI:** 10.1016/j.sopen.2024.02.002

**Published:** 2024-02-19

**Authors:** Rishi Trikha, Logan Laubach, Viraj Sharma, Rachel Thompson, Nicholas Bernthal, Riley J. Williams, Kristofer J. Jones

**Affiliations:** a UCLA Department of Orthopaedic Surgery, 10833 Le Conte Avenue, Los Angeles, CA 90095, USA; b Virginia Commonwealth University, School of Medicine, 1201 E Marshall St #4-100, Richmond, VA 23298, USA; c Sports Medicine and Shoulder Service, Hospital for Special Surgery, 535 East 70th St, New York, NY 10021, USA

**Keywords:** Ethnic diversity, Gender diversity, Trainee diversity, Orthopaedic surgery

## Abstract

**Background:**

There is a lack of physician ethnic and gender diversity amongst surgical specialties. This study analyzes the literature that promotes diversity amongst surgical trainees. Specifically, this study sought to answer (i) how the number of publications regarding diversity in orthopaedic surgery compares to other surgical specialties, (ii) how the number of publications amongst all surgical subspecialties trends over time and (iii) which specific topics regarding diversity are discussed in the surgical literature.

**Methods:**

The Preferred Reporting Items for Systematic Reviews and Meta-Analyses guidelines were used to query articles from PubMed, Web of Science, Embase and the Cumulative Index to Nursing and Allied Health Literature. Broad inclusion criteria for both ethnic and gender diversity of any surgical specialty were utilized.

**Results:**

Our query resulted 1429 publications, of which 408 duplicates were removed, and 701 were excluded on title and abstract screening, leaving 320 to be included. The highest number of related publications was in orthopaedic surgery (*n* = 73) followed by general surgery (*n* = 56). Out of 320 total articles, 260 (81.3 %) were published after 2015, and 56 of 73 (76.7 %) orthopaedic-specific articles were published after 2015.

**Conclusion:**

Orthopaedic surgery published the most about ethnic and gender diversity, however, still remains one of the least diverse surgical specialties. With the recent increase in publications on diversity in surgical training, close attention should be paid to ethnic and gender diversity amongst surgical trainees over the coming years. Should diversity remain stagnant, diversification efforts may need to be restructured to achieve a diverse surgeon workforce.

**Key message:**

Orthopaedic surgery is the surgical subspecialty that publishes the most about trainee ethnic and gender diversity followed by general surgery. With most of this literature being published over the last eight years, it is imperative to pay close attention to the ethnic and gender landscape of the surgeon workforce over the coming years.

## Highlights

• Orthopaedic surgery is the surgical subspecialty that publishes the most about ethnic or gender diversity.
• The number of publications regarding diversity has greatly increased amongst all surgical subspecialties in recent years.
• Literature on diversity focuses on general demographics, pipeline/recruitment and the residency/fellowship application.

## Introduction

Physician ethnic and gender diversity is inextricably linked to patient outcomes, healthcare literacy and access to care in underrepresented patients [[1], [2], [3], [4], [5]]. Despite this, the ethnic and gender disparities prevalent amongst healthcare providers continue to contribute to inequalities in the American healthcare system [2,6,7]. Surgeons are well-known for lacking diversity at the faculty and trainee level throughout all surgical specialties. This is particularly true, however, for orthopaedic surgeons [[8], [9], [10], [11], [12], [13]].

The current United States Census Bureau reports that 59.3 % of the population is White, 18.9 % is Hispanic or Latino, 13.6 % is African American, 6.1 % is Asian, 1.3 % is American Indian, and 0.3 % is Pacific Islander [14]. However, only 6 % of the surgeon workforce is Hispanic or Latino and only 6 % is African American [8]. Women are also underrepresented in surgical field with only 32 % of the surgeon workforce being female [8]. Furthermore, of academicians that rank as full professors, only 2.6 % identify as Hispanic or Latino, 2.2 % as African American and 27.6 % as female [15,16].

Orthopaedic surgery, in particular, is one of the least diverse surgical subspecialties. Only 1.7 % of the American Academy of Orthopaedic Surgeons (AAOS) are Latino, 1.5 % are African American and 86.6 % are White [12]. Racial inequality in orthopaedic surgery is also present in academic research, as only 2 % of NIH funding recipients are African American and only 2 % are Latino [17]. In addition to racial inequality, gender diversity is also lacking in orthopaedic surgery. Despite 57 % of undergraduate students and 51 % of medical students in the United States being female, only 15 % of orthopaedic residents and 7 % of AAOS members are female [[18], [19], [20]]. While there has been a small increase in the percentage of female orthopaedic residents over the past 20 years, this rate of increase is significantly lower when compared to other specialties [11].

As awareness of these disparities becomes more apparent, there has been an increased emphasis on promoting surgeon ethnic and gender diversity at all levels of training [[21], [22], [23], [24]]. While there is both an acute awareness of the lack of diversity in surgical specialties and increased efforts to recruit diverse trainees at all levels of academia [[21], [22], [23], [24], [25]], it remains unclear if either has translated into more research into inequity in surgical training or in a more diversified surgical workforce. Through a review of the literature regarding ethnic and gender diversity amongst trainees throughout all surgical subspecialties, this study sought to answer (i) how the number of publications regarding ethnic or gender diversity in orthopaedic surgery compares to other surgical specialties, (ii) how the number of publications amongst all surgical subspecialties has trended over time and (iii) which specific topics regarding ethnic and gender diversity are discussed in the surgical literature. The authors hypothesize that the number of publications regarding ethnic or gender diversity is highest in the general surgical literature. The authors also hypothesize that the number of these publications has increased in recent years across all surgical subspecialties.

## Methods and search criteria

A review of the literature on ethnic and gender diversity in all surgical subspecialties was performed in accordance with the Preferred Reporting Items for Systematic Reviews and Meta-Analyses (PRISMA) guidelines [26]. Queries of PubMed, Cumulative Index to Nursing and Allied Health Literature (CINAHL), Embase, and the Web of Science databases were performed in April 2022. This search included a combination of keywords and controlled vocabulary for the constructs of surgical subspecialties, residency training, and diversity from the inception of each database until April 1st, 2022.

The Accreditation Council for Graduate Medical Education (ACGME) and the Association of American Medical Colleges (AAMC) were utilized to identify all surgical subspecialties. The PubMed Medical Subject Headings (MeSH) to be included were “Specialties, Surgical”[MeSH] in combination with each individual specialty name “orthopedic, “orthopedic surgery”, “orthopaedicORorthopaedic surgery”, “ENTORotolaryngologyORsurgical critical careORtrauma surgery”, “hand surgery”, “head and neck surgery”, “craniofacial surgery”, “plastic surgery”, “plasticsORcardiothoracicORcardiac surgery”, “general surgery”, “vascular surgery”, “thoracic surgery”, “colon surgery”, “rectal surgery”, “obstetricsORobstetricORgynecologyORneurological surgery”, “neurosurgeryORcolorectal surgery”, “maxillofacial surgery”, “urologyORgynecologicORreconstructive surgery”, “ophthalmologyORophthalmicORotorhinolaryngologyORsurgical oncology”, “surgeryORpediatric surgery.” All terms were searched in the [Title/Abstract] field.

The PubMed MeSH terms “Education, Medical, Graduate”[MeSH] and “Internship and Residency”[MeSH] were used in combination with “residencyORresidentORtrainee”, “fellowORfellowship.” All were searched in the [Title/Abstract] field.

Diversity-related terms were searched as follows: (((“raceORracialORgenderORethnicityORethnicitiesORminorityOR minoritiesORunderrepresented”) AND (“disparityORdisparitiesORdiverseOR diversityORdiversificationOR diverseness”)) OR diverse[Title/Abstract] OR diversity[Title/Abstract]) in the [Title/Abstract] field in order to capture all iterations of gender and ethnic diversity.

This search process is outlined again in Table 1 (Table 1).

**Table 1:** Overview of article query process.

Major term categories	Pubmed MeSH term	Search term
Surgical subspecialty terms	Specialties, Surgical	AND orthopedic OR orthopedic surgery OR orthopaedic OR orthopaedic surgery OR ENT OR otolaryngology OR surgical critical care OR trauma surgery OR hand surgery OR head and neck surgery OR craniofacial surgery OR plastic surgery OR plastics OR cardiothoracic OR cardiac surgery OR general surgery OR vascular surgery OR thoracic surgery OR colon surgery OR rectal surgery OR obstetrics OR obstetric OR gynecology OR neurological surgery OR neurosurgery OR colorectal surgery OR maxillofacial surgery OR urology OR gynecologic OR reconstructive surgery OR ophthalmology OR ophthalmic OR otorhinolaryngology OR surgical oncology OR surgery
Trainee terms	Education, Medical, Graduate	AND (residency OR resident OR trainee OR fellow OR fellowship)
Diversity terms		(race OR racial OR gender OR ethnicity OR ethnicities OR minority OR minorities OR underrepresented) AND (disparity OR disparities OR diverse OR diversity OR diversification OR diverseness)

### Article selection process

The Rayyan Intelligent Systematic Review software was used to screen and track all articles (“Rayyan software”; https://www.rayyan.ai/) [27] with three reviewers screening the title and abstract. Full-text articles were obtained for review if necessary. Any disagreements for inclusion of articles were resolved through discussion between the three reviewers with any disputes being settled by a fourth reviewer. Broad inclusion criteria were utilized to include articles that discussed either ethnic or gender diversity as it pertained specifically to any surgical subspecialty. Articles that exclusively discussed diversity in non-surgical medical fields and those that only discussed diversity related to religion, age, or sexual orientation were excluded.

### Data extraction

The following data were extracted from all articles included in the review: surgical subspecialty, type of diversity (gender, ethnic or both), level of training (medical student, resident or fellow), journal name, date of publication, authors. Any article discussing diversity in multiple surgical subspecialties was counted in its own group (“multiple subspecialties”). This categorization was performed by three authors independently, with any disputes being settled by a fourth reviewer.

### Data analysis

Upon initial review of included articles, each title/abstract was given a brief summary of their respective main aim and conclusion. These summaries were then reviewed by three independent reviewers and categorized into one of the following topics described. The topics were demographics, pipeline/recruitment, application process, training experience, leadership and workplace treatment. If there were any disagreements, a fourth reviewer served as adjudicator, as above. While no well-defined methods currently exist for categorizing orthopaedic surgery diversity literature, the authors felt that each of the included articles fit into these major topics. Articles labeled under the topic of “demographics” discussed program/applicant gender or ethnic diversity at a snapshot or interval of time. Articles characterized as “pipeline/recruitment” discussed programs' efforts to recruit trainees of diverse backgrounds to surgical specialties as well as factors that contribute to trainees selecting a program. Articles characterized in the category of “application process” discussed aspects of the residency or fellowship application process such as letters or recommendation, examinations, interviews or match statistics. Articles labeled under “training experience” discussed the impact of diversity on the medical student, resident or fellow training experience (exposure, mentorship, autonomy, competency, etc.). Articles identified by the topic of “leadership” discussed the diversity of program leadership, such as program directors and department chairs, as well as how this contributed to trainee recruitment. Articles characterized under “workplace treatment” discussed how diversity impacts the workplace experience through topics such as harassment, bias, parental leave or mental health. Descriptive statistics were used to summarize counts and percentages and to examine trends in publication rate over time. The counts for each of the included articles' surgical specialty, year published, topic, and type of diversity were input into Excel (Microsoft Corporation. Version 2303 (2022)) and percentages of each topic were calculated. Each of the included orthopaedic articles were placed into tables displaying the article title, authors, year published, and summary of the article stratified by each of the major aforementioned topics. These can be found in our Supplemental Tables.

Articles were also classified by type of journal that they were published in. These classifications include specialty specific journals, education journals, generic surgical journals and other. “Specialty specific” journals encompass all journals that are tied to a specific surgical subspecialty. “Education” journals were defined as any journal whose primary focus is to publish research pertaining to education at any medical training level. “Generic surgical” journals include journals that do not focus on a particular specialty and where education is not the primary focus of the journal. Other journals are general medical journals or journals that otherwise do not fit into the other journal categories.

## Source of funding

No funding was required for the completion of this project.

## Results

**Fig. 1:**
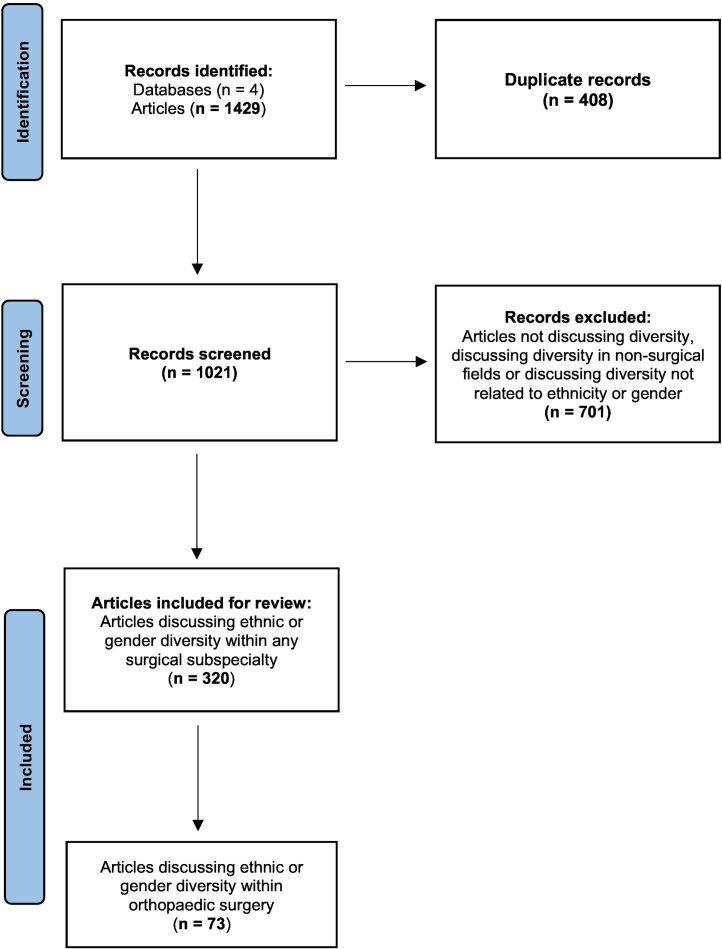
Preferred reporting items for systematic reviews and meta-analysis (PRISMA) flow diagram of identification, screening and inclusion of articles.

**Fig. 2:**
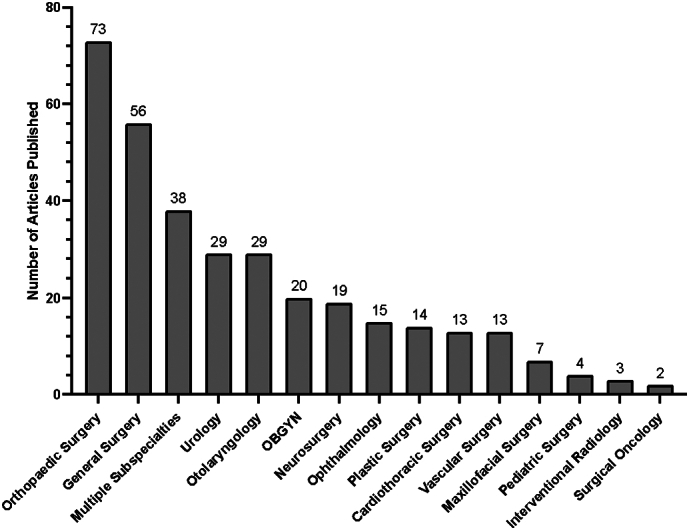
Number of articles regarding ethnic or gender diversity stratified by surgical subspecialty.

The initial query resulted in 1429 total articles. Four hundred and eight duplicates were found and removed. Of the remaining 1021 articles screened, 701 were excluded on screening of the title and abstract, with the remainder of the 320 studies included for review (Fig. 1). After stratifying by surgical subspecialty, orthopaedic surgery was the subspecialty with the largest number of publications regarding ethnic or gender diversity (*n* = 73), followed by general surgery (*n* = 56), urology (*n* = 29) and otolaryngology (n = 29). Notably, 38 articles discussed multiple surgical specialties (Fig. 2).

**Fig. 3:**
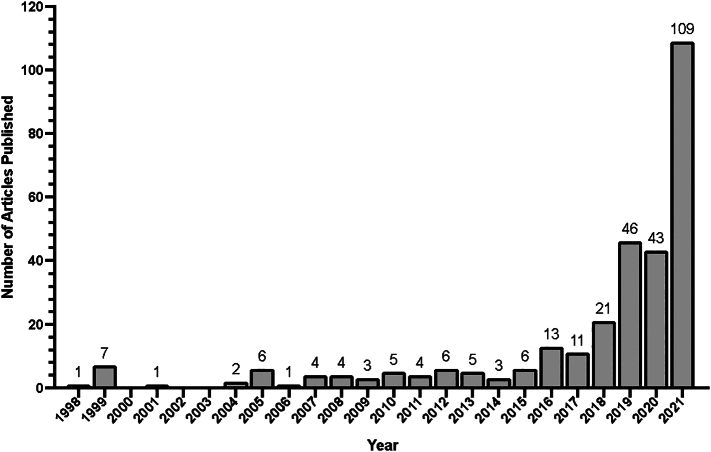
Number of articles regarding ethnic or gender diversity in all surgical subspecialties over time.

**Fig. 4:**
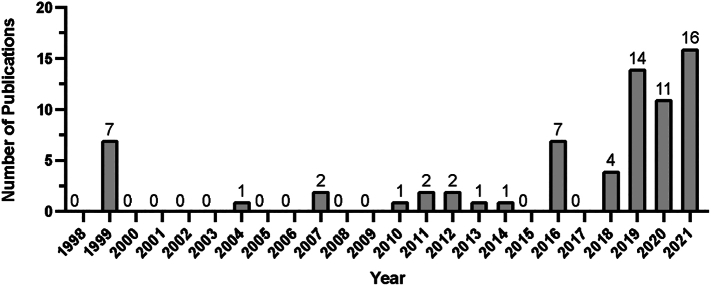
Number of articles regarding ethnic or gender diversity in orthopaedic surgery over time.

Fig. 3 illustrates the yearly breakdown of the 320 studies in all surgical subspecialties dating back to 1998. A total of 109 studies regarding surgical diversity were published in 2021 compared to just 22 studies during a 10-year period from 1998 to 2007 (Fig. 3). Two articles were published prior to 1998 and 17 articles were published in 2022 prior to April 1st, 2022. Two hundred and sixty out of 320 (81.3 %) articles were published after 2015. Fig. 4 demonstrates the number of studies in orthopaedic surgery published by year dating back to 1998 (Fig. 4). No articles were published prior to 1998, and four articles were published in 2022 (prior to April 1st, 2022). Fifty-six out of 73 (76.7 %) articles were published after 2015.

A majority of articles focused solely on gender diversity (*n* = 144; 45.0 %) with fewer articles focusing solely on ethnic diversity (*n* = 39; 12.2 %). A total of 137 (42.8 %) articles analyzed both ethnic and gender diversity. Two hundred and forty-one articles discussed ethnic or gender diversity at the resident level, 42 articles at the fellow level and 37 articles at the medical student level.

**Fig. 5:**
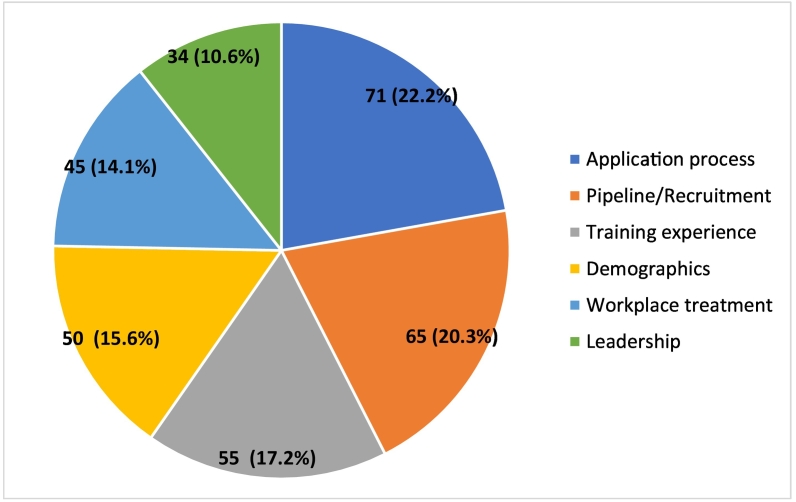
Number of articles discussing diversity in all surgical subspecialties surgery stratified by topic.

**Fig. 6:**
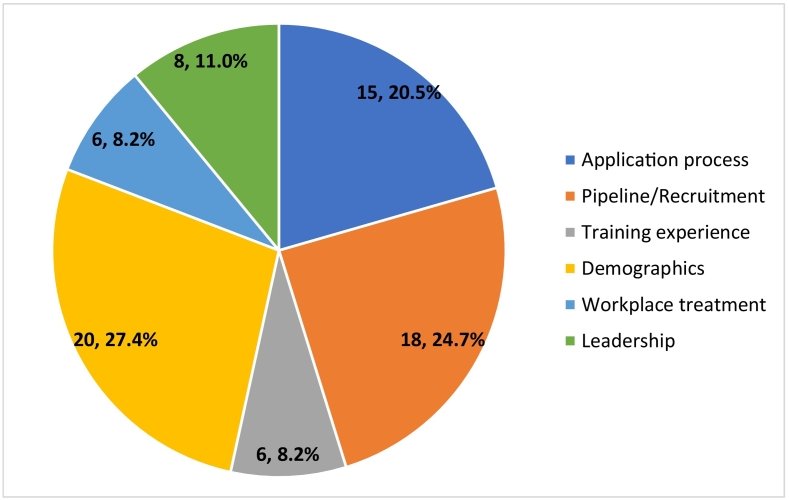
Number of articles discussing diversity in orthopaedic surgery stratified by topic.

After stratifying articles discussing ethnic or gender diversity in all surgical subspecialties by topic, 71 were categorized in the category of “application process,” 65 in “pipeline/recruitment,” 55 in “training experience,” 50 in “demographics,” 45 in “workplace treatment” and 34 in “leadership.” (Fig. 5). After stratifying articles discussing ethnic or gender diversity in orthopaedic surgery by topic, 20 were categorized in the category of “demographics,” 18 in “pipeline/recruitment,” 15 in “application process,” eight in “leadership,” six in “training experience” and six in “workplace treatment.” (Fig. 6). Supplemental Tables 1–6 provide a summary of each article discussing ethnic or gender diversity in orthopaedic surgery stratified by topic (Supplemental Tables 1–6).

Articles were also stratified by journal type. One hundred and sixty articles fell into the category of “specialty specific” journals, with 72 of those articles being in an orthopaedic journal. There were 37 articles in the “education” journal category and 62 in the “generic surgical” journal category. Sixty one articles were classified in the “other” journal category (Table 2).

**Table 2:** Articles stratified by journal type.

Type of journal	Specialty specific (orthopaedic surgery)	Education	Generic surgical	Other
Number of articles	160 (72)	37	62	61

## Discussion

Ethnic and gender diversity amongst surgeons is paramount to improving patient outcomes, improving access to care, optimizing scientific research and enhancing innovation [12,28]. As the importance of diversity becomes increasingly acknowledged, efforts to promote ethnic and gender diversity amongst the physician workforce continue to increase [[21], [22], [23], [24], [25]]. The current review indicates that orthopaedic surgery is the surgical subspecialty with the most publications regarding trainee ethnic and gender diversity. This review also demonstrates that there has been a rapid increase in recent years in the number of articles published regarding trainee ethnic and gender diversity in all surgical subspecialties.

### Number of publications regarding ethnic or gender diversity

Orthopaedic surgery should be applauded for having the most publications regarding trainee ethnic and gender diversity. However, it remains one of the least diverse surgical subspecialties [11,12,17,19,20]. A majority of the orthopaedic literature on trainee diversity focuses on general demographics, pipeline/recruitment efforts as well as the residency/fellowship application process. It is possible that given the majority of this literature focuses on prospective trainee gender and ethnic diversity, there will be correspondent efforts focused on increasing diversification at the trainee level in the upcoming years. It is possible that the number of publications in the orthopaedic literature is related to the number of trainees relative to other specialties. According to 2023 publicly available data, general surgery had the highest number of active residents (1670), followed by obstetrics-gynecology (1503), orthopaedic surgery (899) and ophthalmology (516), urology (383), otolaryngology (373) and neurosurgery (243) [[29], [30], [31]].

### Number of publications in recent years

A study utilizing data from the American Association of Medical Colleges from 2006 to 2015 demonstrated that female representation in orthopaedic surgery residency increased at a significantly lower rate than other specialties. This data also demonstrated that there was actually a decrease in overall diversity amongst orthopaedic residents from 2006 to 2015 [11]. The results of the current study reveal that during that same 10-year period, there were only nine publications about diversity in the orthopaedic surgery literature. It is possible that the number of publications about trainee diversity over a given time period is a marker of diversification efforts at that time, the fruits of which are not realized until a later time period. Recent increases in diversity-related publications may be reflected in upcoming years, but it is imperative that the orthopaedic community continue to monitor the effects of diversification efforts. In fact, a majority of medical schools actively sought to ethnically diversify their student body in 2008 [32]. Despite this, the number of African American male medical school applicants in 1978 was higher than the number of African American male medical school applicants in 2015 [33], further highlighting the importance of monitoring diversification efforts, measuring their effects and changing course if the desired outcomes are not realized.

### Call for targeted diversification efforts

In order to diversify the surgeon workforce, it is important to understand the rationales and motivations for students selecting a surgical specialty. Baldwin et al. demonstrated that female orthopaedic surgery applicants found that early exposure to orthopaedic surgery in medical school was a motivating factor to pursue orthopaedics. In the same study, the largest deterrent for female applicants were programs with minimal female faculty and resident representation [34]. URM students found that the number of URM faculty and residents as well as word of mouth from URM mentors and/or residents were factors affecting their residency selection [35]. Promoting ethnic and gender diversity amongst residents thus requires deliberate effort and may lead to a domino effect with female and URM surgeons attracting female and URM trainees.

It is crucial that diversification efforts are focused, reach their intended audiences and achieve their intended goals. There has been a lot of interest in promoting diversity in medical students in order to diversity the medical workforce [21,23,24]. However, this interest has not translated into increasing surgical trainee diversity. In fact, there has been no difference in the number of underrepresented minorities (URMs) in surgical residencies between 2010 and 2018 [25]. Furthermore, although there are now more female medical students than male, only 33 % of all surgical residents in 2020 were female [19]. Adelani et al. also demonstrated that the number of orthopaedic programs without any URM residents actually increased from 2002 to 2016 [36]. Additionally, the number of URM orthopaedic surgical residents has decreased by roughly 32 % over the past 10 years [11]. The lack of diversity at the resident and medical school level underscores the importance of diversification programs and initiatives directed at the early stages of academia.

One such directed program is the Perry Initiative, which introduces female high school and medical students to orthopaedic surgical techniques. Participants in the initiative are significantly more likely to match into orthopaedic surgery than non-participating women [37,38]. Similarly, orthopaedic programs that participated in the Nth Dimensions program had significantly higher URM faculty and residents [39]. Furthermore, female and URM medical students who completed the Nth Dimensions/American Academy of Orthopaedic Surgeons Orthopaedic Summer Internship Program were significantly more likely to apply to orthopaedic residency [40]. Bernstein et al. also demonstrated that a required medical school musculoskeletal course resulted in almost double the number of female applications to orthopaedic surgery and 34.4 % more applications from URMs [41]. Proven, action-based diversification efforts need to reach students even earlier in their academic career, as female medical students who went into orthopaedic surgery were more likely to report having positive experiences with orthopaedic surgery prior to medical school [42]. Even a one-day program designed to provide female high school students early exposure to orthopaedic surgery increased interest amongst participants [43]. This further highlights the importance of early exposure to diversify the orthopaedic surgeon workforce.

### Limitations

There are several limitations to the current study. Although the number of studies regarding ethnic or gender diversity was reported, there was no internal control for quality of studies. As such, the current study makes no remarks on the quality of these articles as the methodology and level of evidence is quite variable. Furthermore, although ethnic and gender diversity are often linked, they are two separate topics. Given that the goals of this review were to demonstrate a trend in publications as well as subspecialty-specific trends, the authors elected to group articles discussing ethnic and gender diversity together. The subjective nature of the categorization of articles poses an additional possible limitation. To the authors' knowledge, there are no well-validated methods to categorize articles discussing trainee diversity and although there may be overlap between categories, the authors believe that the current categorization largely encompasses most facets of training and thus accept this limitation. Another possible limitation is that there may be more emphasis on trainee diversity in surgical subspecialties with a higher number of surgical residents. This may correlate with the number of publications on the matter. Despite these limitations, this is the first systematic review of its kind to analyze orthopaedic surgical trainee ethnic and gender diversity.

## Conclusion

Orthopaedic surgery is the surgical subspecialty that publishes the most about ethnic and gender diversity amongst trainees with a large uptick in publications in recent years. While literature focusing on diversity amongst trainees is appreciated, efforts and initiatives that directly increase participation at all stages of academia are imperative. With ethnic minorities being projected to comprise the majority of the United States population by 2044 [44], disparities in the surgeon workforce and the resulting consequences in patient care are at risk of exacerbation if considerable action is not taken.

**Specialty specific** journals encompass all journals that are tied to a specific surgical subspecialty.

**Education** journals were defined as any journal whose primary focus is to publish research pertaining to education at any medical training level.

**Generic surgical** journals include journals that do not focus on a particular specialty and where education is not the primary focus of the journal.

**Other** journals are general medical journals or journals that otherwise do not fit into the other journal categories.

## Funding source

None.

## Ethics approval

Not applicable.

## CRediT authorship contribution statement

**Rishi Trikha:** Writing – review & editing, Writing – original draft, Visualization, Validation, Project administration, Methodology, Investigation, Formal analysis, Data curation, Conceptualization. **Logan Laubach:** Writing – original draft, Methodology, Investigation, Formal analysis, Data curation, Conceptualization. **Viraj Sharma:** Writing – original draft, Methodology, Investigation, Formal analysis, Data curation, Conceptualization. **Rachel Thompson:** Writing – review & editing, Supervision, Project administration, Methodology, Investigation, Formal analysis, Data curation, Conceptualization. **Nicholas Bernthal:** Writing – review & editing, Supervision, Project administration, Methodology, Investigation, Formal analysis, Data curation, Conceptualization. **Riley J. Williams:** Writing – review & editing, Validation, Supervision, Project administration, Methodology, Conceptualization. **Kristofer J. Jones:** Writing – review & editing, Writing – original draft, Validation, Supervision, Project administration, Methodology, Formal analysis, Data curation, Conceptualization.

## Declaration of competing interest

All authors have no financial or other conflicts of interest pertinent to this work.

## Appendix A Supplementary data

Supplementary tables Image 1
