# Supplementary material for: Are our actions matching our words? A review of trainee ethnic and gender diversity in orthopaedic surgery
Source: Surg Open Sci. 2024 Feb 19;18:62–9. doi: 10.1016/j.sopen.2024.02.002 (PMC10901127; doi:10.1016/j.sopen.2024.02.002)
Supplement: Supplementary file 1 — Supplementary tables [file mmc1.docx]

**Supplemental Tables Legend:**

**Table 1: Summary of orthopaedic literature categorized as “demographics” discussing program/applicant gender or ethnic diversity at a snapshot or interval of time**

**Table 2: Summary of orthopaedic literature categorized as “pipeline/recruitment” discussing programs’ effort to recruit and expose students/trainees of diverse backgrounds to orthopaedic surgery**

**Table 3: Summary of orthopaedic literature categorized as “application process” discussing aspects of the residency/fellowship application process**

**Table 4: Summary of orthopaedic literature categorized as “leadership” discussing the diversity of program leadership, such as program directors and department chairs, as well as how this contributes to trainee recruitment.**

**Table 5: Summary of orthopaedic literature categorized as “training experience” discussing the impact of diversity on the training experience at the medical school, resident or fellow level**

**Table 6: Summary of orthopaedic literature categorized as “workplace treatment” discussing how diversity impacts the trainee workplace experience**

**Table 1: Summary of orthopaedic literature categorized as “demographics” discussing program/applicant gender or ethnic diversity at a snapshot or interval of time**

| Article Title | Year | Authors | Journal | Summary |
| --- | --- | --- | --- | --- |
| The Distribution of Underrepresented Minorities in U.S. Orthopaedic Surgery Residency Programs. | 2019 | Adelani MA; Harrington MA; Montgomery CO | Journal of Bone and Joint Surgery | The Graduate Medical Education Track database was queried to demonstrate that the number of programs per year with greater than 1 URM resident decreased over time from 2002 to 2016. Furthermore, the number of programs per year without any URM residents increased from 2002 to 2016. |
| Examining Parity among Black and Hispanic Resident Physicians | 2021 | Bennett C.L.; Yiadom M.Y.A.B.; Baker O.; Marsh R.H. AO - Bennett, Christopher L. | Journal of General Internal Medicine | A cross-sectional, retrospective analysis of 11 years of publicly available data demonstrates that Obstetrics/Gynecology, Emergency Medicine, Internal Medicine/Pediatrics, and Orthopedic Surgery had a statistically significant increase in the proportion of Hispanic trainees, but it could take these specialties 35, 54, 61, and 93 years, respectively, to achieve Hispanic representation similar to that of the US population. |
| Has diversity increased in orthopaedic residency programs since 1995? | 2012 | Daniels EW; French K; Murphy LA; Grant RE | Clinical Orthopaedics and Related Research | A questionnaire distributed by email to program coordinators of all orthopaedic residency training programs in the United States showed that the percentage of female orthopaedic surgery residents and female clinical faculty has nearly doubled since 1995. Orthopaedic basic science research faculty, on the other hand, is 83% male and comprised primarily of Caucasians (62%) and Asian/Pacific Islanders (24%). |
| Diversity based on race, ethnicity, and sex between academic orthopaedic surgery and other specialties: a comparative study. | 2010 | Day CS; Lage DE; Ahn CS | Journal of Bone and Joint Surgery | Public registries from 2006 and 2007 showed that there was a significant decrease in the representation of URMs and females from medical schools to orthopaedic residencies (p < 0.001). Females and Asian-Americans were disproportionately underrepresented as full professors. |
| Current diversity in orthopaedics issues of race, ethnicity, and gender | 1999 | England S.P.; Pierce Jr. R.O. | Clinical Orthopaedics and Related Research | The diversity of orthopaedic residents changed minimally between 1983 and 1995. Amongst orthopaedic residents, the percentage of Asian/Pacific Islander men increased from 2.2% to 9.8%, while the percentage of Asian/Pacific Islander women remained the same. The percentage of white women has remained virtually unchanged. |
| Improving diversity in orthopaedic residency programs. | 2007 | Gebhardt MC | Journal of the American Academy of Orthopaedic Surgeons | Discussion regarding how multi-level changes such as expanding K-12 education in minorities, promoting female/minority medical students to orthopaedics, and affirmative action programs would help increase diversity in residency and faculty. |
| A survey of the ethnic and racial distribution in orthopedic residency programs in the United States | 1999 | Grant, RE; Banks, WJ; Alleyne, KR | Journal of the National Medical Association | A questionnaire mailed to chairpersons in 1995 demonstrated that 84.2% of orthopedic residents and fellows were white non-Hispanic, 6.6% were Asian, 3.6% were African American, 2.2% were Native American, 1.2% were Puerto Rican, 0.8% were Mexican American, and 1% were other Hispanic. |
| A Comparison of Applicant and Resident Physician Demographics Among Surgical Subspecialties From 2009 to 2019: Trends in Gender and Underrepresented Minorities in Medicine. | 2021 | Jain A; Nichols G; Tarabishy S; Scomacao I; Herrera FA | Annals of Plastic Surgery | Graduate medical education reports from 2009 to 2019 showed that integrated plastic surgery and otolaryngology surgery had a significantly higher percentage of female residents compared with orthopedic surgery and neurosurgery. Orthopedic surgery and neurosurgery had a significantly higher percentage of African American residents compared with otolaryngology and plastic surgery. |
| Barriers to minorities in the orthopaedic profession. | 1999 | Jiménez RL | Clinical Orthopaedics and Related Research | Discussion highlighting that one third of the United States population is comprised of Latinos, African Americans, and Native Americans. However, only 7% of all orthopaedic surgeons represent these minorities. Misconceptions from minority students, medical school admissions committees, and orthopaedic residency program directors programs may lead to this discrepancy. |
| Pediatric Orthopedic Workforce: A Review of Recent Trends. | 2019 | Minaie A; Shlykov MA; Hosseinzadeh P | Orthopedic Clinics of North America | Discussion demonstrating that pediatric orthopaedics had the highest proportion of female applicants into the field (25%) out of all orthopaedic subspecialties. |
| Revisiting the Gender Gap in Orthopaedic Surgery: Investigating the Relationship Between Orthopaedic Surgery Female Faculty and Female Residency Applicants | 2019 | Munger, AM; Heckmann, N; McKnight, B; Dusch, MN; Hatch, GF; Omid, R | Journal of the American Academy of Orthopaedic Surgeons | Publicly available data from 2005 to 2014 demonstrated that females accounted for 48.7% of medical school graduates, 14.9% of orthopaedic surgery applicants, and 13.2% of full-time orthopaedic surgery faculty. Furthermore, no correlation between the average number of female orthopaedic surgery faculty at an institution and the total number of female orthopaedic surgery applicants from that institution was found, highlighting the complex nature of this gender gap. |
| Racial and ethnic diversity in orthopaedic surgery residency programs. | 2011 | Okike K; Utuk ME; White AA | Journal of Bone and Joint Surgery | Publicly available data showed that during the 1990s and 2000s, representation among orthopaedics increased rapidly for Asians (+4.53% per decade), Hispanics (+1.37% per decade) and African Americans (+0.68% per decade), however orthopaedic surgery was significantly less diverse than all other residency specialties (p < 0.001). |
| Race and ethnic diversity in orthopaedic surgery residency | 2018 | Poon S.; Kiridly D.; Mutawakkil M.; Gecelter R.; Wendolowski S.; Porter R.; Lane L. | Pediatrics | Publicly available data from 2006 to 2014, showed a positive change in orthopaedic resident demographics; a 0.53% increase for African Americans, 2.36% increase for Asian-Americans and 1.72% increase for Hispanic or Latino. |
| Evaluation of Sex, Ethnic, and Racial Diversity Across US ACGME-Accredited Orthopedic Subspecialty Fellowship Programs | 2019 | Poon, S; Kiridly, D; Brown, L; Wendolowski, S; Gecelter, R; Vetere, A; Kline, M; Lane, L | Orthopedics | Publicly available data showed that between 2006 and 2015, 68.5% of orthopaedic fellows were white, 17.4% were Asian, 7.4% were Hispanic, 4.3% were Black, 0.21% were Native Hawaiian/Pacific Islander, and 0.08% were American Indian/Alaskan Native. Racial and ethnic minority representation did not increase over time. 12.9% of fellows were females and proportionally increased with female residents. |
| Workforce Analysis of Spine Surgeons Involved with Neurological and Orthopedic Surgery Residency Training. | 2019 | Post A.F.; Dai J.B.; Li A.Y.; Maniya A.Y.; Haider S.; Sobotka S.; Germano I.M.; Choudhri T.F. | World Neurosurgery | Faculty members of neurological surgery and orthopedic surgery residency training programs were reviewed to demonstrate that of the female spine surgeons, those with neurosurgical training (64.44%) nearly doubled the number with orthopedic training (35.56%). |
| Geographic Differences in Sex and Racial Distributions Among Orthopaedic Surgery Residencies: Programs in the South Less Likely to Train Women and Minorities | 2019 | Rajani, R; Haghshenas, V; Abalihi, N; Tavakoli, EM; Zelle, BA | Journal of the American Academy of Orthopaedic Surgeons Global Research and Reviews | Data for active residents in the United States from 2013 to 2014 were obtained from the American Medical Association with program regions divided into Northeast, Midwest, South and West. Orthopaedic residency programs in the South were less likely to include women or racial minorities as compared to the West and Northeast (p = 0.034 and p < 0.001, respectively). |
| Racial, ethnic, and gender diversity and the resident operative experience - How can the Academic Orthopaedic Society shape the future of orthopaedic surgery? | 1999 | Simon, MA | Clinical Orthopaedics and Related Research | A 1996 report demonstrated that African Americans and Hispanics make up only 2% each of orthopaedic faculty and women represent only 8% of orthopaedic faculty. A further discussion regarding that membership of the Academic Orthopaedic Society can change this composition by providing mentoring of present medical students. |
| Women and minorities in orthopaedic residency programs. | 2007 | Templeton K; Wood VJ; Haynes R | Journal of the American Academy of Orthopaedic Surgeons | Publicly available data on medical student demographics and orthopaedic surgery residencies demonstrated that the percentages of women and URMs including African Americans, Hispanics, Asian/Pacific Islanders were statistically significantly lower (p < 0.001 for all groups) among those training in orthopaedic residency programs compared with those same groups entering and graduating from medical school. |
| African Americans and women in orthopaedic residency. The Johns Hopkins experience. | 1999 | Thomas CL | Clinical Orthopaedics and Related Research | At a single institution, African Americans have comprised 22.9% of all residents admitted through the match program between enrollment years 1992 and 1998, and women have comprised 14.3%. This is in contrast with African Americans and females comprising only 1.5% and 2.3%, respectively, of all United States orthopaedists. |
| The uneven distribution of women in orthopaedic surgery resident training programs in the United States. | 2012 | Van Heest AE; Agel J | Journal of Bone and Joint Surgery | Publicly available data between 2004-2009 showed that 45 orthopaedic residency programs had no female residents during at least one year. More than 50 programs had an average of <10% female residents over the 5 years analyzed demonstrating that significant differences in the representation of women exist among orthopaedic residency training programs in the United States. |

**Table 2: Summary of orthopaedic literature categorized as “pipeline/recruitment” discussing programs’ effort to recruit and expose students/trainees of diverse backgrounds to orthopaedic surgery**

| Article Title | Year | Authors | Journal | Summary |
| --- | --- | --- | --- | --- |
| Gender Disparities Within US Army Orthopedic Surgery: A Preliminary Report | 2018 | Daniels CM; Dworak TC; Anderson AB; Brelin AM; Nesti LJ; McKay PL; Gwinn DE | Military Medicine | A census of US Army active-duty orthopaedic surgeons showed that 10.3% of this surgeon population are women. Discusses how increasing diversity can expand the future talent pool. |
| Inspiring Women in Engineering and Medicine: The Impact of a One-Day STEM Experience on High School Females' Attitude Toward Male-Dominant Professions | 2021 | DiCosmo AM; Isch E; Coyner K | Journal of Surgical Education | In response to the statement “I am interested in orthopedics,” 127/475 (27%, p < 0.01) high school students who participated in an Inspiring Women in Orthopedics and Engineering immersive 1-day program changed their answer from not being interested to being interested. |
| Breakout session: Diversity, cultural competence, and patient trust. | 2011 | Dy CJ; Nelson CL | Clinical Orthopaedics and Related Research | Discussion regarding the relative success of improving the diversity of the orthopaedic surgery workforce amongst female residents but illustrating that there has been no improvement amongst African American and Hispanic residents. Early exposure pipeline programs are important for recruiting diverse orthopedic applicants. |
| What is the trend in representation of women and underrepresented minorities in orthopaedic surgery residency? | 2021 | Haffner M.R.; Van B.W.; Wick J.B.; Le H.V. | Clinical Orthopaedics and Related Research | Orthopaedic surgery had the lowest representation of women residents every year, with women residents comprising 16% of residency classes in 2020. During the past decade there was only a small increase in the representation of women orthopaedic surgery, while the representation of people from URM groups did not change. This is contrary to general surgery which has substantially reduced gender and ethnic disparities. |
| The Perry Initiative Impact on Gender Diversity Within Orthopedic Education | 2021 | Harbold D; Dearolf L; Buckley J; Lattanza L | Current Reviews in Musculoskeletal Medicine | Discusses how the Perry Initiative has reached over 12,000 women in high school and medical school. Of the program participants eligible to match to a residency program 20% matched into orthopaedic surgery. |
| The Perry Initiative Medical Student Outreach Program Recruits Women into Orthopaedic Residency | 2016 | Lattanza LL; Meszaros-Dearolf L; O'Connor MI; Ladd A; Bucha A; Trauth-Nare A; Buckley JM | Clinical Orthopaedics and Related Research | 31% of women in the first graduating class of the Medial Student Outreach Program (MSOP) to promote gender diversity in orthopaedic surgery matched into orthopaedic surgery. The Perry Initiative's MSOP positively influenced women to choose orthopaedic surgery as a profession. |
| Improving Sexual, Racial, and Ethnic Diversity in Orthopedics: An Imperative | 2020 | Lin JS; Lattanza LL; Weber KL; Balch Samora J | Orthopedics | The lack of diversity in orthopaedic surgery stems from the pipeline as female and underrepresented minorities are not applying to orthopaedic surgery |
| Impact of a Musculoskeletal Clerkship on Orthopedic Surgery Applicant Diversity. | 2016 | London DA; Calfee RP; Boyer MI | American Journal of Orthopedics | A required 1 month third-year medical student orthopaedic rotation led to an 81% relative increase in female applicants and a 101% relative increase in underrepresented minority applicants. |
| Can a Strategic Pipeline Initiative Increase the Number of Women and Underrepresented Minorities in Orthopaedic Surgery? | 2016 | Mason BS; Ross W; Ortega G; Chambers MC; Parks ML | Clinical Orthopaedics and Related Research | For women, completion in an Orthopaedic Summer Internship was associated with increased odds of applying to an orthopaedic surgical residency (OR: 51.3, 95% CI: 21.1-122.0; p < 0.001). For underrepresented minorities, an Orthopaedic Summer Internship completion was also associated with increased odds of applying to an orthopaedic surgical residency (OR: 14.5, 95% CI: 7.3-27.5, p < 0.001). |
| Women in Orthopaedics: How Understanding Implicit Bias Can Help Your Practice | 2020 | Mulcahey MK; Van Heest AE; Weber K | Instructional Course Lectures | Provide an overview of the current status of women in orthopaedics and bring awareness to implicit bias. Women comprise roughly 50% of medical students, however only 14% of orthopaedic residents. |
| A track record of diversity: Medical schools ranked by successful black applicants to orthopaedic residencies | 2022 | Nsekpong T.B.; Ode G.; Purcell K.; Randhawa R.; Dixon T.; Stewart G.; Mkorombindo T.; Pinto M.; Mesfin A.; Kemp A.; Bhanat E.; Thimothee J.; Williams B.A.; Bolarinwa S.; Brooks J.T. AO - Nsekpong, Tyler B.;Brooks, Jaysson T. | Journal of the National Medical Association | The J. Robert Gladden Orthopaedic Society database was queried to identify medical schools that have successfully matched black applicants into orthopaedic surgery. |
| Medical School Experiences Shape Women Students' Interest in Orthopaedic Surgery | 2016 | O'Connor MI | Clinical Orthopaedics and Related Research | Ten-year review of women in orthopaedic surgery that demonstrates successful recruitment can be achieved by early exposure and access to role models. |
| Current Trends in Sex, Race, and Ethnic Diversity in Orthopaedic Surgery Residency | 2019 | Poon S; Kiridly D; Mutawakkil M; Wendolowski S; Gecelter R; Kline M; Lane LB | Journal of the American Academy of Orthopaedic Surgeons | AAMC data from 2006 to 2015 showed that female representation increased from 10.9% to 14.4%, however the rate of this increase was significantly lower when compared to other specialties. This data also showed that there was no significant change in African American or Asian American representation over the study period. |
| Racial Diversity in Orthopedic Surgery | 2019 | Ramirez RN; Franklin CC | Orthopedic Clinics of North America | Demonstrates lack of diversity by utilizing data from the AAOS and ACGME as well as discussing how improving diversity would improve patient care and also attract top medical students |
| Where Are the Women in Orthopaedic Surgery? | 2016 | Rohde RS; Wolf JM; Adams JE | Clinical Orthopaedics and Related Research | Survey of 232 practicing female orthopedic surgeons showing the most common reason for choosing orthopaedic surgery was enjoyment of manual tasks and the most common reasons for not choosing orthopaedic surgery was perceived inability to have a good work/life balance, the perception that too much physical strength is required and lack of strong mentorship |
| Analysis of Factors Related to the Sex Diversity of Orthopaedic Residency Programs in the United States. | 2018 | Sobel AD; Cox RM; Ashinsky B; Eberson CP; Mulcahey MK | Journal of Bone and Joint Surgery | Orthopaedic programs with more female residents had more female faculty members per program (p=0.001), a higher percentage of faculty who were female (p<0.001), more female associate professors (p<0.001), more women in leadership positions (p<0.0010.) |
| What Proportion of Women Who Received Funding to Attend a Ruth Jackson Orthopaedic Society Meeting Pursued a Career in Orthopaedics? | 2019 | Vajapey S; Cannada LK; Samora JB | Clinical Orthopaedics and Related Research | 80% of female medical students who were offered a scholarship to attend the Ruth Jackson Orthopaedic Society (RJOS) annual meeting are currently practicing orthopedic surgery or are in an orthopaedic surgery residency program suggesting that early influenced exposure to the field can contribute to gender diversity within the field |
| Gender Diversity in Orthopedic Surgery: We All Know It's Lacking, but Why? | 2020 | Van Heest A | Iowa Orthopaedic Journal | ACGME records showed that 12 residency programs have no women trainees, highlighting the uneven distribution of females across residency programs |

**Table 3: Summary of orthopaedic literature categorized as “application process” discussing aspects of the residency/fellowship application process**

| Article Title | Year | Authors | Journal | Summary |
| --- | --- | --- | --- | --- |
| Does Removing the Photograph and Name Change the Reviewer's Perception of Orthopaedic Residency Applicants? | 2021 | Caldwell LS; Garcia-Fleury I; An Q; Lawler EA | The Journal of the American Academy of Orthopaedic Surgeons | During the 2018-2019 orthopaedic residency application cycle at a single institution, applicant photographs and names were removed from the reviewer’s perception. After propensity matching, this resulted in a URM candidate being 2.5 times more likely to get an interview than a non-URM candidate. |
| Orthopedic Surgery Residency Application Process in 2020 - Has Diversity been Affected? | 2021 | Caldwell LS; Lawler EA | Iowa Orthopaedic Journal | A retrospective review at a single institution demonstrated no significant differences in URM or female applicants being invited to interview for an orthopaedic residency program (p=0.66 and p=0.63 respectively) between the 2018 application cycle and the 2020 application cycle which was a virtual rotation and interview platform. |
| Women in Orthopaedic Fellowships: What Is Their Match Rate, and What Specialties Do They Choose? | 2016 | Cannada LK | Clinical Orthopaedics and Related Research | Available fellowship match data demonstrated that female fellowship applicants had a higher proportion of match success when compared with men (96% vs 81%, p < 0.001). Pediatric orthopaedic fellowships had the highest proportion of women (25%) followed by foot and ankle (14%), with spine surgery having the lowest (3%). |
| The Impact of the COVID-19 Pandemic on Orthopaedic Surgery Residency Applicants During the 2021 Residency Match Cycle in the United States. | 2020 | Danford NC; Crutchfield C; Aiyer A; Jobin CM; Levine WN; Lynch TS | Journal of the American Academy of Orthopaedic Surgeons | Women and URM rising fourth year medical students felt like they had less opportunities to be adequately exposed to orthopaedic surgery due to the pandemic. Subsequently, women were “less likely” to apply to orthopaedic surgery (14.9% versus 5.5% of men, p < 0.001). Black/African American also said that they were “less likely” to apply (16.9% compared with 8.8 of non-Hispanic White, p < 0.001). |
| A Focused Gap Year Program in Orthopaedic Research: An 18-Year Experience | 2020 | Egol KA; Shields CN; Errico T; Iorio R; Jazrawi L; Strauss E; Rokito A; Zuckerman JD | Journal of the American Academy of Orthopaedic Surgeons | Students who complete a gap year dedicated to orthopaedic research at a single institution matched at a higher rate than the national average despite a lower step score. This research cohort had a higher percentage of women (23%) and minorities (40%) than the proportion of women and minority practicing orthopaedic surgeons. |
| Women and men in orthopaedics. | 2021 | Errani C; Tsukamoto S; Kido A; Yoneda A; Bondi A; Zora F; Soucacos F; Mavrogenis AF | Société Internationale de Chirurgie Orthopédique et de Traumatologie (SICOT) | Literature review demonstrating that the existence of a gender gap within orthopaedic surgery may be multifactorial and lies in both organizational and individual factors. |
| Factors in Orthopaedic Residency Decision-making for Female Applicants: A Cross-sectional Study. | 2020 | Goss ML; McNutt SE; Hallan DR; Bible JE | Journal of the American Academy of Orthopaedic Surgeons | A survey sent to female orthopaedic residents asking them to score their most important factors when selecting an orthopaedic surgery residency demonstrated that the most important factors were not sex-related factors such as happiness, camaraderie, variety/number of cases and surgical/clinical experience. Least important factors included sex diversity of faculty and residents, number of female residents and attitudes toward maternity leave. |
| Current Orthopaedic Residency Letters of Recommendation Are Not Biased by Gender of Applicant. | 2021 | Lipa SA; Greene NE; Le HV; White AA 3rd; Gebhardt MC; Dyer GSM | Journal of Bone and Joint Surgery | Letters of recommendation for orthopaedic residency applicants at a single institution over two application cycles showed that word count was, on average, longer for female applicants. Ability and participation in athletics were also found more frequently in letters of recommendation for females. |
| Applicant Fit and Diversity in the Orthopaedic Surgery Residency Selection Process: Defining and Melding to Create a More Diverse and Stronger Residency Program. | 2020 | Modest JM; Cruz AI Jr; Daniels AH; Lemme NJ; Eberson CP | Journal of Bone and Joint Surgery | Editorial discussing how a comprehensive understanding of "fit" and "culture” add to an orthopaedic residency program can promote the drive for diversity. |
| Orthopaedic Faculty and Resident Sex Diversity Are Associated with the Orthopaedic Residency Application Rate of Female Medical Students. | 2019 | Okike K; Phillips DP; Swart E; O'Connor MI | Journal of Bone and Joint Surgery | Data provided by the Association of American Medical Colleges from 2014 to 2017 showed that 1.98% of women graduating from medical school applied to an orthopaedic surgery residency program. Women who attended medical school at institutions with high orthopaedic faculty sex diversity were more likely to apply for residency in orthopaedics (p = 0.023) |
| Race, But Not Gender, Is Associated with Admissions into Orthopaedic Residency Programs. | 2020 | Poon SC; Nellans K; Gorroochurn P; Chahine NO | Clinical Orthopaedics and Related Research | Hierarchical logistic regression models utilizing publicly available data and that controlled for metrics of academic performance demonstrated that applicants from Asian (OR 0.78 [95% CI 0.67 to 0.92]), Black (OR 0.63 [95% CI 0.51 to 0.77], Hispanic (OR 0.48 [95% CI 0.36 to 0.65]), or other race groups (OR 0.65 [95% CI 0.55 to 0.77]) had lower odds of admission into residency compared with white applicants. |
| Underrepresented Minority Applicants Are Competitive for Orthopaedic Surgery Residency Programs, but Enter Residency at Lower Rates | 2019 | Poon, S; Nellans, K; Rothman, A; Crabb, RAL; Wendolowski, SF; Kiridly, D; Gecelter, R; Gorroochurn, P; Chahine, NO | Journal of the American Academy of Orthopaedic Surgeons | Minority applicants to orthopaedic residency programs comprised 29% of applicants. 61% of minority applicants were accepted into an orthopaedic residency versus 73% of White applicants (p < 0.0001). White applicants and matriculated candidates had higher Step 2 Clinical Knowledge scores and higher odds of Alpha Omega Alpha membership. |
| Race- and Gender-Based Differences in Descriptions of Applicants in the Letters of Recommendation for Orthopaedic Surgery Residency. | 2020 | Powers A; Gerull KM; Rothman R; Klein SA; Wright RW; Dy CJ | Journal of Bone and Joint Surgery | The use of a standardized letter of recommendation reduced the use of certain words that imply gender or race-based bias when compared to the traditional letter of recommendation at a single institution. This suggests the use of a standardized letter of recommendation could potentially reduce gender and race-based bias. |
| Gender, Race, Age, Allopathic Degree, Board Score, and Research Experience Among Applicants Matching to General and Orthopedic Surgery Residencies, 2015-2019 | 2022 | Sutherland, M; Sanchez, C; Baroutjian, A; Ali, A; McKenney, M; Elkbuli, A | American Surgeon | Publicly available data showed that male and female residency applicants matched into general surgery at rates of 23.0% and 29.2%, respectively. Male and female residency applicants matched into orthopaedic surgery at rates of 55.2% and 56.2%, respectively. Women match into general surgery and orthopaedic surgery at higher rates than men but comprise disproportionately lower numbers of applicants. |
| Is There Unconscious Bias in the Orthopaedic Residency Interview Selection Process? | 2022 | Webber C.R.J.; Davie R.; Herzwurm Z.; Whitehead J.; Pare D.W.; Homlar K.C. AO - Webber, Colton R.J.; Whitehead, Jonathon; Pare, Daniel W. | Journal of Surgical Education | Orthopaedic residency applicants for the 2019-2020 match cycle at a single institution were initially reviewed and scored by faculty members. Applications were then redacted of all identifying information (including race and gender) and rescored by all faculty members. Pre-redaction scores were significantly higher than post-redaction scores in white applicants, indicating a possible implicit bias. |

**Table 4: Summary of orthopaedic literature categorized as “leadership” discussing the diversity of program leadership, such as program directors and department chairs, as well as how this contributes to trainee recruitment.**

| Article Title | Year | Authors | Journal | Summary |
| --- | --- | --- | --- | --- |
| The role of gender in academic productivity, impact, and leadership among academic spine surgeons | 2022 | Agaronnik N.; Xiong G.X.; Uzosike A.; Crawford A.M.; Lightsey H.M.; Simpson A.K.; Schoenfeld A.J. AO - Xiong, Grace X. | Spine Journal | The academic productivity of all spine faculty across orthopaedic residencies and fellowships and neurosurgical fellowships were analyzed. Women comprised only 5.6% of this faculty. Furthermore, women had 40% fewer total publications (p = 0.025), h-indices approximately 5 units lower than men (p = 0.006) and approximately half the high-impact senior author publications (p = 0.007) than men. |
| Orthopaedic Foot and Ankle Surgery Fellowship Directors Are Typically White Men in Their Early 50s With Strong Achievements in Research | 2021 | Elahi M.A.; Moore M.L.; Doan M.K.; Pollock J.R.; Hassebrock J.D.; Makovicka J.L.; Brinkman J.C.; Patel K.A. | Arthroscopy, Sports Medicine, and Rehabilitation | Data for each foot and ankle fellowship director via publicly available means were collected and showed that 93.6% were male, 89.4% were white, and all had relatively high research productivity. |
| Analysis of Gender Diversity Within Hand Surgery Fellowship Programs | 2021 | Grandizio, LC; Pavis, EJ; Hayes, DS; Young, A; Klena, JC | Journal of Hand Surgery | Data for each hand surgery fellowship program were obtained and demonstrated that of the 89 hand surgery programs evaluated, 36 (60%) had at least 1 female faculty member. For the 849 prior fellows identified, 213 (25%) were female, and 79% of programs had at least 1 female fellow. Hand programs led by a female director did not have a higher percentage of prior female fellows compared to programs led by a male director (26% vs 25%). |
| Gender Differences in Program Factors Important to Applicants When Evaluating Orthopaedic Surgery Residency Programs. | 2019 | Kroin E; Garbarski D; Shimomura A; Romano J; Schiff A; Wu K | Journal of Graduate Medical Education | All applicants to a single orthopaedic surgery residency program in the 2017 Match filled out a survey which demonstrated that female applicants rated the presence of female and minority residents and faculty as well as program reputation for gender and racial/ethnic diversity higher than male applicants. |
| A Cross-Sectional Evaluation of the Successful Minority Applicant to Orthopaedic Surgery Residency Programs. | 2021 | Malige A; Wells L; Brooks JT; Mesfin A; Talwar D; Leska T; Klevins H | Journal of Racial and Ethnic Health Disparities | An anonymous survey of URM students applying to orthopaedic residency in 2020 showed that 88.9% of URM applicants viewed the presence or absence of URM faculty as an important factor in their rank list and 87.5% reported that this distinction was meaningful to their orthopaedic candidacy. |
| Diversity in orthopaedics | 1999 | Mankin HJ | Clinical Orthopaedics and Related Research | Discussion about how women, African Americans and Hispanics have fewer opportunities to enter the orthopaedic community. These groups who are already part of the orthopaedic community have fewer opportunities to become contributing members of orthopaedic programs in the United States. |
| Barriers to Increasing Diversity in Orthopaedics: The Residency Program Perspective. | 2020 | McDonald TC; Drake LC; Replogle WH; Graves ML; Brooks JT | Journal of Bone and Joint Surgery | A survey distributed to program directors of all orthopaedic surgery residency programs between 2018 and 2019 showed that the most commonly stated barriers to increasing diversity within orthopaedic surgery programs were: "We do not have enough minority faculty, which may deter the applicants" (69.3%), "We consistently rank minority applicants high but can never seem to match them" (56%), and "Not enough minorities are applying to our program" (54.7%). |
| Orthopaedic Faculty and Resident Racial/Ethnic Diversity is Associated with the Orthopaedic Application Rate Among Underrepresented Minority Medical Students | 2020 | Okike, K; Phillips, DP; Johnson, WA; O'Connor, MI | The Journal of the American Academy of Orthopaedic Surgeons | Data provided by the Association of American Medical Colleges demonstrated that 5.4% of all URM students who graduated from medical school from 2013 to 2017 applied to an orthopaedic residency program. URM students who attended medical school at institutions with high URM representation on the orthopaedic faculty were more likely to apply in orthopaedics (p = 0.02), as were URM students at institutions with high URM representation in the residency program (p < 0.001). |

**Table 5: Summary of orthopaedic literature categorized as “training experience” discussing the impact of diversity on the training experience at the medical school, resident or fellow level**

| Article Title | Year | Authors | Journal | Summary |
| --- | --- | --- | --- | --- |
| The relationship between required medical school instruction in musculoskeletal medicine and application rates to orthopaedic surgery residency programs. | 2004 | Bernstein J; Dicaprio MR; Mehta S | Journal of Bone and Joint Surgery | Medical school survey along with residency application data demonstrated that the rate of application to orthopaedic surgery residency programs for female students was 2.0% for those who had required musculoskeletal courses during medical school compared with 1.1% of females who did not have a dedicated musculoskeletal course. URM students who had a required musculoskeletal course applied at a rate of 8.2% compared to 6.1% for students that did not have a required course. |
| Improvement of Orthopedic Residency Programs and Diversity: Dilemmas and Challenges, an International Perspective. | 2019 | Fayaz HC; Smith RM; Ebrahimzadeh MH; Pape HC; Parvizi J; Saleh KJ; Stahl JP; Zeichen J; Kellam JF; Mortazavi J; Rajgopal A; Dahiya V; Zinser W; Reznik L; Shubnyakov I; Pećina M; Jupiter JB | Archives of Bone and Joint Surgery | A questionnaire sent to residency programs in 10 countries on four different continents indicated that standardizing structure and requirements for orthopaedic residency training across countries may help increase diversity amongst trainees. |
| Objective Test Scores Throughout Orthopedic Surgery Residency Suggest Disparities in Training Experience | 2021 | Foster, N; Price, M; Bettger, JP; Goodwin, CR; Erickson, M | Journal of Surgical Education | The performance of all orthopaedic residents at a single institution demonstrated that females with similar step 1 scores to males had lower orthopedic in-service exam scores as well as American Board of Orthopedic Surgery Part 1 scores possibly due to their experiences during residency. |
| Residents' perceptions of sex diversity in orthopaedic surgery. | 2013 | Hill JF; Yule A; Zurakowski D; Day CS | Journal of Bone and Joint Surgery | Publicly available data as well as results from a survey emailed to 2629 orthopaedic residents demonstrated that female representation in both orthopaedic and general surgery has increased since 1968, but it has increased significantly faster in general surgery than in orthopaedic surgery (p < 0.0001). |
| Mentorship in Orthopedics: A National Survey of Orthopedic Surgery Residents. | 2018 | Oladeji LO; Ponce BA; Worley JR; Keeney JA | Journal of Surgical Education | A questionnaire distributed to orthopedic residents demonstrated that only two-thirds of orthopedic residency programs have mentorship programs despite 95% of residents valuing the importance of mentorship. URMs were less likely to have multiple mentors and more likely to be dissatisfied with the quality of mentorship in residency. Females were more likely to pursue mentorship on their own. |
| How Do Medical Students Perceive Diversity in Orthopaedic Surgery, and How Do Their Perceptions Change After an Orthopaedic Clinical Rotation? | 2021 | Rahman R.; Zhang B.; Humbyrd C.J.; LaPorte D. | Clinical Orthopaedics and Related Research | A survey of students from 27 US medical schools who had completed an orthopaedic rotation demonstrated that medical students of demographic backgrounds who are not the majority in orthopaedics generally perceived that orthopaedic surgery is less diverse and inclusive than do their counterparts in majority groups, however these views often changed after a clinical orthopaedic rotation. |

**Table 6: Summary of orthopaedic literature categorized as “workplace treatment” discussing how diversity impacts the trainee workplace experience**

| Article Title | Year | Authors | Journal | Summary |
| --- | --- | --- | --- | --- |
| Gender Differences in Pediatric Orthopaedics: What Are the Implications for the Future Workforce? | 2016 | Amoli, Marielle; Flynn, John; Edmonds, Eric; Glotzbecker, Michael; Kelly, Derek; Sawyer, Jeffrey; Amoli, Marielle A; Flynn, John M; Edmonds, Eric W; Glotzbecker, Michael P; Kelly, Derek M; Sawyer, Jeffrey R | Clinical Orthopaedics and Related Research | Survey sent to members of the Pediatric Orthopaedic Society of North America that demonstrated that among new graduates, women are more likely to choose academic practice (women: 13 of 18 [72%], men: 21 of 44 [48%], p < 0.001) whereas men are more likely to choose private practice (men: 14 of 44 [32‰], women: one of 18 [6%], p < 0.001). Men were also more likely to report having job offers before starting their fellowship (men: 24 of 44 [54%], women: eight of 18 [44%], p = 0.042) |
| Pregnancy and Parental Leave During Orthopaedic Surgery Residency. | 2021 | Compton J; Hajewski CJ; Pugely AJ | Iowa Orthopaedic Journal | Discussion about a potential barrier to the recruitment and retention of female orthopedic surgeons in the controversies surrounding pregnancy and parental leave during residency training, current policies, health considerations and perceptions of parental leave and potential future recommendations. |
| Barriers for Medical Students with Underrepresented Identities Considering Orthopaedic Surgery Careers: A Qualitative Investigation | 2021 | Gerull K.M.; Parameswaran P.; Cogsil T.; Jeffe D.; Salles A.; Cipriano C.A. | Journal of the American College of Surgeons | Medical students from four Midwest medical schools were interviewed (16 of whom were women, and 12 of whom were URMs) about perceived barriers for medical students with underrepresented identities. Perceived barriers for minority medical students’ perceptions of barriers to applying to orthopedics largely fell into two categories: (1) barriers in the residency application/selection process and (2) fears about a perpetual lack of belonging. |
| What factors influence applicants' rankings of orthopaedic surgery residency programs in the National Resident Matching Program? | 2014 | Huntington WP; Haines N; Patt JC | Clinical Orthopaedics and Related Research | All orthopaedic residency applicants to a single institution were asked to rank various factors that may have affected their rank list. Women weighed their personal interactions and a program's proximity to family and friends more heavily when determining a rank list. 68% of women eliminated residency programs from their options based on perceived sex biases as compared to less than 1% of men who eliminated programs for the same reason. |
| Barriers to minority participation in the orthopaedic profession. Personal perspectives. | 1999 | Phillips PJ | Clinical Orthopaedics and Related Research | Discussion highlighting that institutional support, mentorship, and recruitment are vital for minority applicant success as well as the establishment of a pathway to an orthopaedic career. |
| True Grit in Leadership: 2018 AOA Critical Issues Symposium Addressing Grit, Sex Inequality, and Underrepresented Minorities in Orthopaedics. | 2019 | Samora, Julie Balch; Ficke, James R.; Mehta, Samir; Weber, Kristy | Journal of Bone and Joint Surgery | Discussion regarding "grit" and how important a trait it is in order to combat implicit biases and microaggressions within orthopaedics. Further discussion regarding the need for the orthopaedic community to work on creating a more welcoming culture to increase diversity within the field |
